# Investigation of the Thermal and Hydrolytic Degradation of Polylactide during Autoclave Foaming

**DOI:** 10.3390/polym13162624

**Published:** 2021-08-06

**Authors:** Julia Dreier, Christian Brütting, Holger Ruckdäschel, Volker Altstädt, Christian Bonten

**Affiliations:** 1 Institut für Kunststofftechnik, University of Stuttgart, Pfaffenwaldring 32, 70569 Stuttgart, Germany; julia.dreier@ikt.uni-stuttgart.de; 2 Department of Polymer Engineering, University of Bayreuth, Universitätsstraße 30, 95477 Bayreuth, Germany; christian.bruetting@uni-bayreuth.de (C.B.); ruckdaeschel@uni-bayreuth.de (H.R.); volker.altstaedt@uni-bayreuth.de (V.A.)

**Keywords:** polylactide, biofoam, hydrolysis, degradation

## Abstract

Polylactide (PLA) is one of the most important bioplastics worldwide and thus represents a good potential substitute for bead foams made of the fossil-based Polystyrene (PS). However, foaming of PLA comes with a few challenges. One disadvantage of commercially available PLA is its low melt strength and elongation properties, which play an important role in foaming. As a polyester, PLA is also very sensitive to thermal and hydrolytic degradation. Possibilities to overcome these disadvantages can be found in literature, but improving the properties for foaming of PLA as well as the degradation behavior during foaming have not been investigated yet. In this study, reactive extrusion on a twin-screw extruder is used to modify PLA in order to increase the melt strength and to protect it against thermal degradation and hydrolysis. PLA foams are produced in an already known process from the literature and the influence of the modifiers on the properties is estimated. The results show that it is possible to enhance the foaming properties of PLA and to protect it against hydrolysis at the same time.

## 1. Introduction

Degradation describes any type of mechanism in which there is a reduction in molecular weight and consequently a shortening of the polymer chains. These include hydrolysis, enzymatic oxidation, photooxidation and auto-oxidation. Mechanical and thermal as well as UV radiation-induced degradation can also occur. All these processes take place without the presence of microorganisms, which is why they are called abiotic degradation processes. This is their difference from biodegradation, which involves microorganisms. The abiotic degradation processes can lead to fragmentation and the formation of small microparticles, which in turn can be metabolized by microorganisms [1,2].

**Figure 1:**
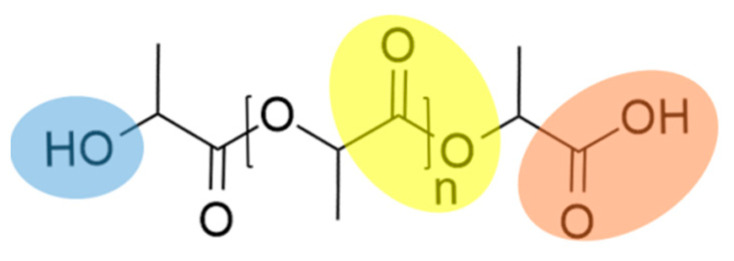
Chemical structure of PLA with its functional groups.

PLA, as with many other bioplastics, is very sensitive to thermal and hydrolytic degradation, which is characteristic of all polyesters. In this process, cleavage takes place at the hydrolyzable groups, such as esters, by water molecules. The structural formula of PLA is shown below in Figure 1, with the functional groups color-coded.

In hydrolysis, a distinction must be made between two different types, acidic and basic ester hydrolysis. Both reactions can occur in PLA and it must be noted whether the reaction takes place within the chain or at the end groups and under which conditions. Hydrolysis is influenced by several parameters, including on the one hand the prevailing environmental conditions such as water activity, temperature, pH and time [3,4]. On the other hand, the degree of crystallization, molar mass, size and geometry of the samples, stereo complex formation, number of acid end groups and hydrophobicity also play a crucial role [5,6,7,8]. A special feature in PLA is also the occurrence of the so-called autocatalyzed hydrolysis. The mechanism is similar to that of acid ester hydrolysis. The proton of the carboxyl group catalyzes the hydrolysis reaction. The proton activates the carbonyl group and makes it more susceptible to attack by water molecules. Under neutral conditions, a slower degradation takes place and in alkaline media, a faster degradation takes place than in acidic environments [1,4,9]. Hydrolysis often occurs during processing (for example extrusion or injection molding) under the influence of high temperatures. One way to counteract this is to pre-dry the PLA pellets very well before processing or to add stabilizers during processing [6,7,10]. However, hydrolysis can occur not only at high temperatures, but also at relatively moderate temperatures around 60 °C and under the influence of increased humidity, such as under industrial compost conditions [11]. 

Hydrolytic degradation does not necessarily end in complete decomposition of the material. However, it must be considered in order to identify and minimize downgrading of the polymer properties. The literature contains numerous papers and patents that counteract undesirable degradation with chemical modifiers [3,6,7,8,10,12,13,14,15,16,17]. The substance classes epoxides, carbodiimides and phosphorous acid esters have shown the most promise to date [10,13]. Most of these modifiers react with the end groups of PLA to inhibit the hydrolysis. The determination of the acid value is a method to verify possible reactions between modifiers and PLA and degradation through processing. Although there are some ways to prevent the hydrolysis of PLA, to the best of our knowledge, this was not investigated for foaming. Especially in bead foaming, PLA has to undergo different processing steps, such as compounding, foaming and welding. 

Often, biopolymers such as PLA are said to be potential alternatives in packaging applications. Here, expanded PS (EPS) and expanded Polypropylene (EPP) are the market leaders due to their possible complex geometries compared with their low densities. In order to compete with these materials, PLA bead foams have to be made and fused together. Standau et al. [18] showed different ways of producing bead foams. As an example, expanded PLA (EPLA) can be made by a stirring autoclave process described by Nofar [19]. This process is characterized by a water-polymer mixture which is processed at temperatures far above 100°C, which leads to a tremendous degradation and therefore a loss in mechanical stability. To be competitive with polyolefine bead foams, the used polylactides need to be modified in order to prevent degradation during processing.

As a conclusion, PLA suffers from hydrolytic degradation during processing. Therefore, several modifiers have been used to increase the molecular structure and prevent the PLA from degradation during processing. Modified samples have been processed in a stirring autoclave process according to the literature to evaluate the stabilizing effect of the modifiers.

## 2. Experimental

### 2.1. Materials

In this study, PLA Ingeo 2003 D from NatureWorks Ltd. (Minnetonka, USA) was modified with two different chemical modifiers. Dicumyl peroxide (DCUP) from Sigma Aldrich was used as a melt strength enhancer and poly carbodiimide from Lanxess, Cologne, Germany as a hydrolysis stabilizer. Both modifiers were incorporated by reactive extrusion with a ZSK 26 twin screw extruder (Coperion GmbH, Stuttgart, Germany). The concentrations were selected based on pre-trials with 0.1 and 0.2 wt% for the peroxide and 0.5 wt% for the poly carbodiimide. 

### 2.2. Methods

#### 2.2.1. Rheological Investigation

Using a Discovery HR-2 plate-to-plate rheometer from TA Instruments, USA (plate diameter 25 mm; plate spacing 1 mm), the linear viscoelastic deformation behavior of PLA and the modified grades was determined. Prior to measurement, the materials were dried overnight at 40 °C in a vacuum oven. The viscosity measurements were then carried out under a nitrogen atmosphere. To determine the thermal stability and to investigate if the chemical reactions were completed, time-sweep measurements were performed at a constant angular velocity of 1 rad/s, a shear stress amplitude of 5 % and a measurement temperature of 180 °C. The measurement time was 1800 s. A frequency sweep was performed to determine the viscoelastic flow behavior. Here, at a shear stress amplitude of 5% and a temperature of 180 °C, the angular velocity is varied from 500 to 0.01 rad/s.

#### 2.2.2. Size Exlcusion Chromatography (SEC)

An Agilent 1260 (Agilent, Waldbronn, Germany), equipped with a PSS-SDV precolumn (10 μm) 50 mm × 8 mm and three linear PSS-SDV columns (10 μm) 300 mm × 8 mm (with pore sizes of 103 Å, 105 Å and 106 Å), from PSS (Mainz, Germany) was used to determine the molar mass. Measurements were made in chloroform at a temperature of 30 °C and a flow rate of 1.0 mL/min. Using a PSS SECurtiy 1260 differential detector (PSS, Mainz, Germany), the molar mass could be calculated. In order to investigate the influence of the modification and the chemical constitution, GPC measurements were performed using a PSS DVD 1260 viscosity detector (PSS, Mainz, Germany). 

#### 2.2.3. Acid Value Determination

The acid number was determined according to EN ISO 2114:2000 to verify whether the acid end group had been modified by reactive extrusion. For the subsequent determination of the acid number, 3 or 6 g PLA were dissolved in 100 mL chloroform (Carl Roth, Karlsruhe/Germany) and stirred for at least three hours. The glass electrode (type 6.0229.100 from Methrom (Filderstadt, Germany)) was then placed in the analysis vessel. While stirring, the methanolic KOH solution was added stepwise (0.5 mL steps or 0.2 mL steps around the equivalence point) and the voltage value of the potentiometer (“AL15” from Tintometer GmbH—Lovibond Water Testing, Dortmund, Germany) was noted. The measurement was stopped as soon as the voltage value of the potentiometer changed only slightly after the addition of the methanolic KOH solution. The potentiometer “AL15” was used (Tintometer GmbH—Lovibond Water Testing, Dortmund, Germany). 

#### 2.2.4. Foaming

A self-made stirring autoclave was used for processing the polymer granules. Here, the materials were processed as it is known for EPLA bead foams in the literature [19]. Deionized water and the pellets were put in the autoclave. Afterwards, it was sealed and a pressure of 50 bar of CO_2_ was applied. The setup was heated up to a temperature of 120 °C, saturated for 30 min and released out of the autoclave. The gained materials after this processing were used to evaluate the degradation behavior of the samples.

## 3. Results

### 3.1. SEC Results

**Figure 2:**
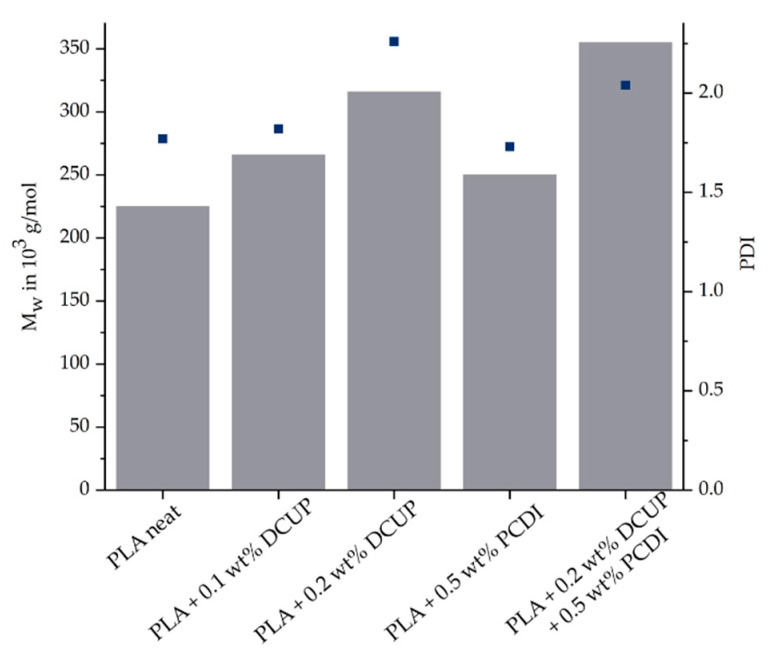
SEC-Results of all PLA compounds.

The used PLA was modified on a twin-screw extruder with DCUP (0.1 wt%/0.2 wt%) and PDCI (0.5 wt%). After the reactive modification, the effectiveness of the modifiers was verified. One way to investigate this is by the method of SEC, which can be used to determine the molar mass and a molar mass distribution. The principle is based on the separation of molecules according to their molecular size. Figure 2 shows the molar mass and polydispersity index (PDI) of all produced compounds. 

The organic peroxide led, as well as the PDCI, to an increased molar mass. As the amount of DCUP increases, this effect is enhanced. The addition of PCDI leads also to an increase in molar mass but is less pronounced than DCUP. The reason why the molar mass does not increase that much is that PCDI only reacts with the end groups of PLA. The combination of DCUP and PCDI showed the highest increase in molar mass. The results show on the one hand that a successful modification took place during the reactive extrusion on the twin-screw extruder and thus a chemical reaction between the two components has occurred. Furthermore, the increase in molar mass indicates a change in the linear structure of the PLA, which will be considered in more detail in the following rheological investigations.

### 3.2. Rheological Investigation

**Figure 3:**
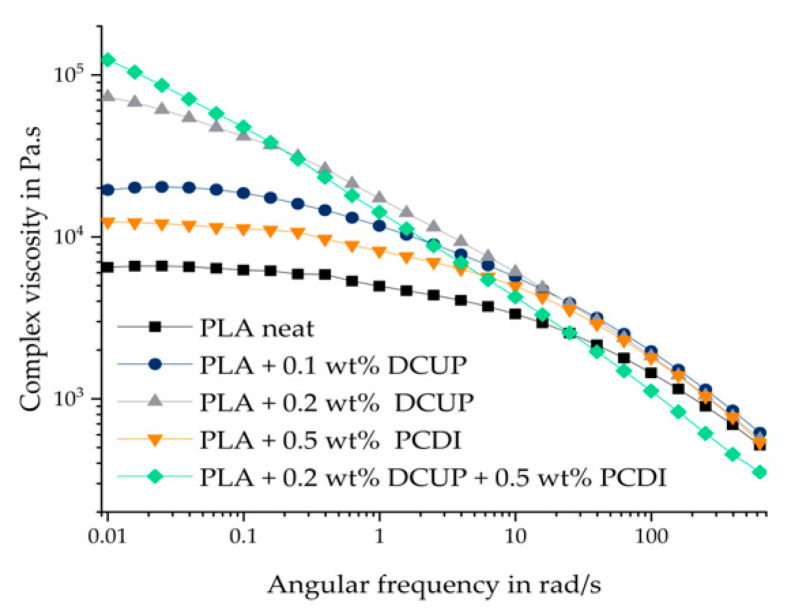
Rheological measurements of the PLA compounds.

Figure 3 shows the measured frequency sweeps of the unmodified PLA compared to the modified ones. 

All materials show a typical shear thinning behavior. Pure, unmodified PLA shows a chain progression typical for linear polymers. Modification with DCUP leads to an increase in zero viscosity, but the curves still show typical shear thinning behavior and form a Newtonian plateau in the low-frequency range, except PLA with 0.2. wt% DCUP and 0.5 wt% PCDI. With higher amount of DCUP, a higher zero shear viscosity is observed. The increase in viscosity can be explained by the change in the molecular chain structure of the modified PLA. DCUP decomposes during reactive extrusion to form radicals that can attack the PLA chain leading to a chain extension or/and branching. It has already been described in the literature that DCUP can lead to branching or cross-linking of polymer chains [12]. The longer chains and branching in the modified PLA result in entanglements that act like a physical network, leading to longer relaxation times. This leads to the fact that the Newtonian range is only indicated at low frequencies. The addition of 0.2 wt% DCUP is more effective than that of 0.1 wt% because a higher number of possible reactive groups are available which leads to an amount of extensions. PCDI was added as a hydrolysis stabilizer. As seen with the SEC results before, the addition of 0.5 wt% PCDI leads to an increase in molar mass und therefore also to an increase in the complex viscosity compared to neat PLA. Consequently, the combination of DCUP with PCDI results in the highest complex viscosity of all materials and to a change in the curve flow due to a complementation of the chain extension and the prevention to degradation.

**Figure 4:**
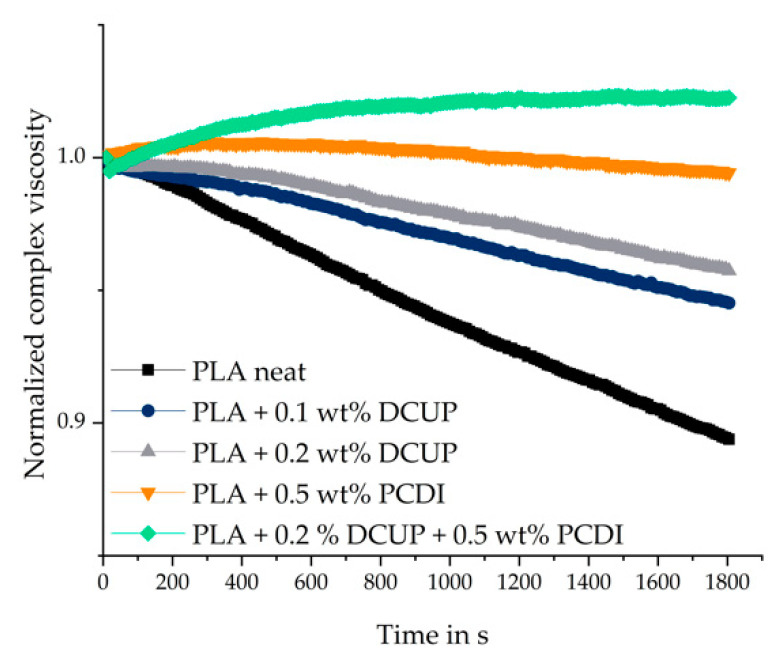
Normalized time-dependent viscosity of the compounds.

Timesweep measurements were used to observe the thermal stability. The thermal stability is crucial for the processing, especially for the foaming process of polymers. In addition, information is obtained about the conversion of the chemical reaction during the reactive extrusion. Degradation reactions as well as post-reactions are generally undesirable and should be avoided or at least taken into account. The normalized complex viscosity as a function of time is shown in Figure 4.

The time sweep was done for 30 min at isothermal temperature of 180 °C for all investigated compounds. It can be seen that the neat PLA has the highest degree of degradation, followed by the different peroxide-containing compounds. The PCDI stabilized material and the modified stabilized modifications show the highest relative viscosity indicating no or even a low degradation during the timesweep. After the first 400 s, for all materials except those with PCDI, the normalized complex viscosity decreases due to degradation processes. The use of PCDI led to the desired stabilization process. However, only a slight decrease in complex viscosity for the PLA with 0.5 wt% PCDI can be observed after 30 min. Additionally, the reaction regarding the chain extension proceeded completely and no post-reactions could be observed. The combination of DCUP and PCDI has the highest complex viscosity and is stable over the time of 1800 s. 

### 3.3. Acid Value Determination 

The determination of the acid value is a method to verify if chemical reactions at the end groups of PLA occurred. Table 1 shows the results of the acid value of the different PLA compounds before and after the foaming. 

The higher the acid value, the higher the amount of carboxyl end groups of PLA and therefore more possibilities for a hydrolytic reaction are given. PCDI can react with these end groups and lead to a small acid value. This means that PCDI is capturing the carboxyl end groups, which hinders the hydrolytic reaction. The results before indicated that a reaction between PLA and PCDI happened. The acid values before foaming were determined after the compounding step. The addition of 0.5 wt% PCDI leads to the smallest acid value. It confirms the other results and the expected reaction of PLA and PCDI. The combination of both modifiers has a higher acid value of 1.15 but it is still in a range where hardly any end groups exist in the whole polymer chains. As a comparison the neat PLA showed an acid value of about 18.8 mmol/Kg. The conditions during the foaming led for all materials to an increase of the acid value indicating a chain scission during the processing. This is not surprising, looking at the processing conditions (PLA pellets in water at 120 °C for 30 min). As it is known from literature, hydrolysis of PLA takes place around 60 °C and under humidity. Therefore, it can be concluded that the processing conditions for the foaming contain the ideal surroundings for hydrolysis. 

## 4. Conclusions

PLA was successfully modified via reactive extrusion. By using 0.2 wt% of an organic peroxide, the molar mass and complex viscosity could be increased. To prevent PLA from undergoing hydrolysis, a stabilizer was used. To receive good foaming properties as well as a thermally and hydrolytically stable PLA, the two modifiers were combined. The combination of 0.2 wt% DCUP and 0.5 wt% PCDI leads to the highest molar mass and the best thermal stabilization. By using the acid value determination, it could be shown that the stabilizer reacts with the end groups of PLA and that several processing steps such as extrusion and foaming can lead to a degradation of PLA. As a result, it is indispensable to prevent PLA from hydrolysis to ensure that there is no downgrade of the properties of PLA. In future works, different modifiers and stabilizers could be investigated and also the following processing steps of bead foams, such as welding. 

## Figures and Tables

**Table 1:** Determined acid values in mmol/kg.

Compound	Acid Value before Foaming	Acid Value after Foaming
PLA neat	18.78 ± 0.04	30.60 ± 0.13
PLA + 0.1 wt% DCUP	20.80 ± 0.25	31.87 ± 1.20
PLA + 0.2 wt% DCUP	26.63 ± 0.19	34.83 ± 0.31
PLA + 0.5 wt% PCDI	0.72 ± 0.03	2.03 ± 0.98
PLA + 0.2 wt% DCUP + 0.5 wt% PCDI	1.15 ± 0.20	5.67 ± 0.31

## Data Availability

Not applicable.
